# The Practical Implications of Re-Referencing in ERP Studies: The Case of N400 in the Picture–Word Verification Task

**DOI:** 10.3390/diagnostics15020156

**Published:** 2025-01-11

**Authors:** Vojislav Jovanović, Igor Petrušić, Vanja Ković, Andrej M. Savić

**Affiliations:** 1 Laboratory for Neurocognition and Applied Cognition, Department of Psychology, Faculty of Philosophy, University of Belgrade, 11000 Belgrade, Serbia; 2 Laboratory for Advanced Analysis of Neuroimages, Faculty of Physical Chemistry, University of Belgrade, 11000 Belgrade, Serbia; 3 Science and Research Centre, School of Electrical Engineering, University of Belgrade, 11000 Belgrade, Serbia

**Keywords:** re-referencing, good scientific practice (GSP), event-related potentials (ERP), N400

## Abstract

**Background:** The selection of an optimal referencing method in event-related potential (ERP) research has been a long-standing debate, as it can significantly influence results and lead to data misinterpretation. Such misinterpretation can produce flawed scientific conclusions, like the inaccurate localization of neural processes, and in practical applications, such as using ERPs as biomarkers in medicine, it may result in incorrect diagnoses or ineffective treatments. In line with the development and advancement of good scientific practice (GSP) in ERP research, this study sought to address several questions regarding the most suitable digital reference for investigating the N400 ERP component. **Methods:** The study was conducted on 17 neurotypical participants. Based on previous research, the references evaluated included the common average reference (AVE), mean earlobe reference (EARS), left mastoid reference (L), mean mastoids reference (MM), neutral infinity reference (REST), and vertex reference (VERT). **Results:** The results showed that all digital references, except for VERT, successfully elicited the centroparietal N400 effect in the picture–word verification task. The AVE referencing method showed the most optimal set of metrics in terms of effect size and localization, although it also produced the smallest difference waves. The most similar topographic dynamics in the N400 window were observed between the AVE and REST referencing methods. **Conclusions:** As the most optimal regions of interest (ROI) for the picture–word elicited N400 effect, nine electrode sites spanning from superior frontocentral to parietal regions were identified, showing consistent effects across all referencing methods except VERT.

## 1. Introduction

Maintaining and improving good scientific practice (GSP) in the field of event-related potentials (ERP) is important, especially given the increasing interdisciplinarity and the growing number of studies utilizing this technique. There have been several major updates in guidelines and standards in electroencephalography (EEG) and magnetoencephalography (MEG), with the most recent by Niso and colleagues [1] not only addressing various issues in different research stages, but also considering GSP in light of ongoing social and ethical challenges. Since the ERP technique is based on the EEG method, it is highly influenced by various factors related to the electrodes, such as their number, location, and reference, just to name a few. Guidelines regarding electrodes indicate that referential recordings are the most optimal in ERP studies, including clearly specified references [2]. Referential montages, which are also called monopolar, are based on the principle that each electrode is connected to the same reference that can be some other scalp electrode, or the average of the signal at all electrodes [3]. Choosing the ERP reference is an important issue that can significantly affect amplitude values and signal polarity, and can shift the overall amplitude of the topography [4]. There is no ideal solution for the most effective reference, since it would require a point with zero or constant potential, which our body cannot provide [5]. There have been various attempts to solve or circumvent this problem, such as choosing the sites with, assumingly, minimal activity (such as earlobes, the tip of the nose, the nasion, mastoids, noncephalic locations, etc.) or trying to model the ideal reference through mathematical calculation, using the common average reference (AVE) [6] or the neutral infinity reference, also known as the reference electrode standardization technique (REST) [7]. Although the AVE is usually regarded as the best referencing option [8,9,10,11], it is dependent on the number and locations of electrodes in the average [12], and should be avoided in analyses that involve a small number of scalp sites [13]. Another disadvantage of the AVE is the difficulty of comparing ERP waveforms and distributions across studies and laboratories [5]. AVE and REST are currently considered superior to other references in ERP research [14,15,16,17,18,19], but both show problems in dealing with electrode density and electrode coverage [20]. Traditionally used references, such as the ears, the nose or mastoids, are also problematic for various reasons, including the influence of cerebrospinal fluid (CSF) pathways, skull holes, and the contributions of underside cortical sources [13].

One of the most researched and widely used ERP components is the negative wave that peaks around 400 milliseconds poststimulus, called the N400. Although typically elicited in experiments that involve some type of language-related expectancy violation, it is believed that N400 represents deeper levels of information processing, contributing to the processing of meaning [21]. Impairment of the N400 is observed in various diseases and conditions, including Alzheimer’s disease [22,23,24], Autism Spectrum Disorder [25,26,27], mild cognitive impairment (MCI) [28,29], Parkinson’s disease [30,31,32], epilepsy [33,34], schizophrenia [35,36], and dyslexia [37]. However, many ERP studies yield inconsistent or conflicting results, which can, in part, be attributed to differences in procedures and researcher choices during signal acquisition and data processing. For example, the concept of “researcher degrees of freedom” suggests that experimenters can analyze their data in multiple ways [38], and this variability is even greater in ERP research [39]. As the N400 represents a promising candidate for a reliable medical biomarker in diagnostics and therapy evaluation, producing valid and reliable results is of great importance.

Evidence from fMRI and MEG studies indicate that the activity of multiple brain regions is related with the N400 effect, with an emphasis on the left temporal cortex and inferior frontal and parietal regions [40,41,42]. Regarding scalp distribution, it is known that the N400 effect is most evident at the centroparietal sites; that is, when an ear or mastoid reference is used, N400 negativity is at its maximum, over superior central and parietal areas [21,43]. One of the earliest N400 studies showing how the results can be affected by various references included mastoid sites (individually and as an average) and the sternum to the seventh cervical vertebra as a noncephalic reference [44]. In this study, focusing on asymmetric brain potentials, an experiment with visually sequentially presented words was used, with an emphasis on experimental conditions that included homophonic words. It was found that the activity at the right mastoid reference site (R) significantly affected obtained results, leaving the left mastoid (L) as a better referencing choice for this kind of experiment. A more recent study indicated that the semantic violation-evoked N400 effect obtained in antonym task is most prominent using the averaged linked mastoid reference (LM), followed by REST and AVE referencing, respectively [45]. As expected, authors found no differences regarding the scalp distribution of N400 between the various reference approaches. Nevertheless, authors point out that, although the LM produces larger amplitudes than the REST and AVE, it can be susceptible to brain activation, thus recommending the REST as the more objective and reference-free approach. A systematic review of papers that investigated picture-evoked N400 showed that most preferred reference used in ERP analysis involved a mastoid or earlobe reference, with the AVE being the second most common approach [46]. Interestingly, some of the referencing solutions were quite odd, like the vertex (VERT). That solution probably relies on an early EEG recording tradition, but still endures to this day despite clear recommendations against it, considering the N400 distribution [5]. As a conclusion, the mean mastoid reference (MM) was indicated to be a reference of choice, since it was the most frequently used a mastoid reference, and allows easier results comparison between studies [46]. Although the MM and mean earlobes reference (EARS) are considered more suitable for N400 recording than individual mastoid or earlobe locations, due their asymmetry resilience [47], there has been evidence of significant influence of MM and L on various properties of obtained data, such as a power spectra shift, EEG coherence, and default mode network connectivity [16]. Nevertheless, the MM is considered by some researchers as a typically used solution in N400 research that is better than the AVE, since it produces larger N400 [48]. Kappenman et al. [49] proposed that the average of the P9 and P10 electrode sites can actually serve as a better choice than the MM, since they provide cleaner signals not only in case of N400, but also for recording mismatch negativity (MMN), N2pc, P300, lateralized readiness potential (LRP) and error-related negativity (ERN). This solution is somewhat unconventional in the scope of the previous ERP tradition, and is yet to be evaluated in future research. In addition to practical issues, there are also terminological problems regarding using MM as a reference. First, most of the ERP studies use the terms “linked mastoids” and “linked earlobes” as synonyms, covering both terms with the same LM abbreviation as in Huang et al. [50] and Yao et al. [51]. Second, the “linked mastoid” term is also used by researchers to refer to both physically linking electrodes and the average of physically separate sites [46]. As we believe that clearly defining and separating these terms is important, in the rest of the text, we will try to avoid terminological ambiguities wherever possible.

It is no secret that the literature on optimal referencing methods in EEG/ERP research is both extensive and long-standing [12,52,53,54]. However, important referencing information is often scattered across numerous articles, making it time-consuming to investigate and decide on the most suitable referencing choice. This task becomes even more complicated when considering that different experimental designs may require different references. Given that most ERP studies focus on one or several specific ERP components, it would be beneficial for researchers to have a practical guide tailored to the component of interest, saving them the time of browsing through various studies. Furthermore, the existence of previous guidelines and findings does not guarantee their automatic implementation in similar research designs. Despite the wealth of findings related to the N400, there remains significant diversity and inconsistency in both the choice of referencing methods and recording locations [46].

The main goal of this study is to improve GSP within N400 research by addressing several questions related to the selection of a digital reference, a crucial step in data preprocessing. It also seeks to expand insights into issues that profoundly influence data quality and result interpretation. First, we examined the influence of commonly used referencing choices on real ERP data, using a priori selected electrodes in the time window that is most optimal for N400 research [21,46]. Second, we aimed to identify the most optimal scalp locations for recording the N400 effect, independent of the reference, or at least resilient enough to maintain the effect despite different referencing choices. Finally, we aimed to shed light on the benefits, limitations, and nuanced considerations of the referencing methods explored in this study, recognizing that our commentary is neither exhaustive nor inclusive of every possible aspect.

To address these questions, we conducted a simple picture–word verification task, applying the same preprocessing routine before re-referencing and averaging, similar to the approach used in previous studies [15]. Our selection of digital references was based on the findings of Šoškić et al. [46], which highlighted several commonly used referencing options: the mean mastoids reference, also known as the average linked mastoids reference (MM), the common average reference (AVE), the mean earlobes reference (EARS), and the left mastoid reference (L). We also included the REST in our study, as it is currently considered one of the most favorable referencing options. Lastly, although it is rarely used, we also included the vertex reference (VERT), to illustrate the effects of using a reference near the region of interest. It is important to note that, in this study, we used the terms ‘mean mastoid’ and ‘mean earlobes’, rather than the more commonly used ‘linked mastoids’ and ‘linked earlobes’, since those terms can be ambiguous, and suggest both physically linked and physically separate sites [5,46]. To our knowledge, no previous studies have directly compared the influence of these referencing choices on the N400 component in the picture–word task. Additionally, no study has specifically investigated the differences between MM and EARS as distinct referencing choices for the N400 component in this task. Our recent work was focused on ERP protocols for identifying potential biomarkers of neurological disorders [55,56,57]. This work aims to contribute to the standardization of ERP recording and analysis protocols, enhancing both basic research practices and practical applications, including clinical diagnostics.

## 2. Materials and Methods

### 2.1. Participants

The G*Power 3.1 software was used to calculate the minimum sample size for this study [58]. Considering the effect size of 0.25, statistical power of 0.95, significance level of 0.05 and the minimum of 40 measurements per experimental situation, a total sample size of 10 participants was required. Data from 17 participants, aged from 23 to 53 years (M = 33.53, *SD* = 8.49) with normal or corrected-to-normal vision was analyzed. As this study was part of a larger project, the participants were carefully selected to represent a neurotypical population. Specifically, individuals with a history of head trauma or neurological or psychiatric disorders, or those taking medications that could affect the results, were excluded. These criteria ensured that the data collected during the experiments accurately reflected the target population. Some concerns about our sample may stem from its relatively wide age range, given the well-documented decline in N400 parameters with advancing age [59]. However, to mitigate age-related effects, this study will focus on the mean amplitude calculated over a specific time range (also referred to as the “mean in window” approach). This measure has demonstrated greater reliability and robustness against various influences, making it a more practical choice compared to other amplitude measures, such as peak amplitude [5]. This study adhered to ethical guidelines and received approval from the Scientific Ethics Committee of the Clinical Center of Serbia and the Neurology Clinic (reference number: 23–690). Written informed consent was obtained from all participants before the study.

### 2.2. Stimuli

The stimuli consisted of 60 photographs depicting easily recognizable and commonly encountered objects, which were resized to fit a box of 400 × 400 pixels, and presented on a light gray background in the middle of the screen subtending the visual angle of about 10°. Each of the images was associated with the pairing word that was either related to the picture (name of the presented object), or unrelated to the picture (name of some other object), resulting in two experimental conditions (congruent vs. incongruent).

### 2.3. Procedure

**Figure 1:**
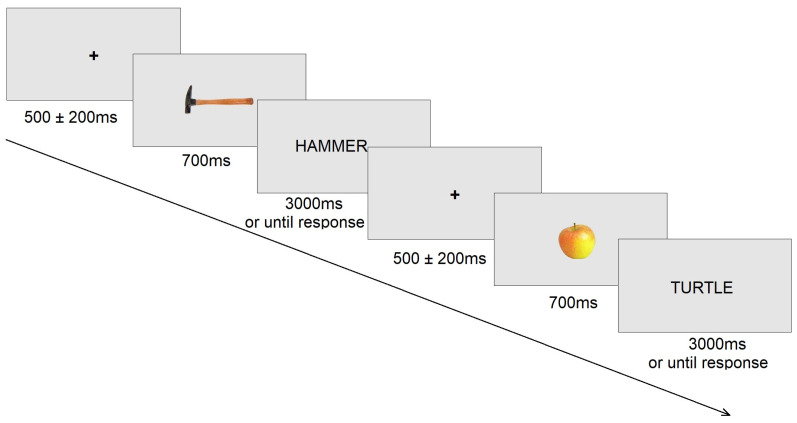
Example of experimental trial.

Participants were seated in a dimly lighted room that was electrically shielded. Trials were presented via OpenSesame 3.3.9 software [60] on a 17-inch CRT monitor at a distance of 60 cm from the participant. Each trial consisted of a fixation cross that appeared in the center of the screen, for the jittered time range between 300 and 700 ms that varied from trial to trial. Next, a picture of the object appeared for 700 ms, followed by the target word which remained on the screen for three seconds or until a response, as shown in Figure 1. Participants were asked to respond as quickly and as accurately as possible, clicking the left mouse button for the picture–word match and the right mouse button for the picture–word mismatch. Sixty congruent and incongruent picture–word pairs were presented to each of the participants, comprising a total of 120 trials. The presentation order was randomized for each participant.

### 2.4. EEG Recording and ERP Processing

Continuous EEG signals were recorded from the 35 scalp sites, according to the international 10/20 standard, with sampling rate of 1000 Hz. The following sites were recorded: Fp1, Fp2, F7, F8, FT9, FT10, T7, T8, F3, Fz, F4, FC5, FC6, FC1, FC2, FCz, C3, Cz, C4, CP5, CP6, CP1, CP2, P3, Pz, P4, TP9, TP10, P7, P8, PO9, PO10, O1, Oz, and O2. The activity of both earlobes (A1 and A2) was also recorded, with the ground electrode positioned at the AFz location. During the experiment, electrode impedance levels were maintained below 5 kΩ. Offline signal processing was performed using EEGLAB [61] and MATLAB software (Version R2023a). The EEG signal was downsampled to 256 Hz, and filtered using a second order IIR Butterworth filter (passband edges: 0.2–10 Hz, cutoff frequency: −6 dB, 12dB/oct roll-off), according to the recent recommendations made for mean amplitude measurement in N400 research [62]. After filtering, any bad channels and portions of continuous data containing high levels of noise were manually removed. Next, independent component analysis (ICA) was conducted using the FastICA picard.m algorithm. Components identified by ICLabel with a probability of 0.9 as eye, muscle, or heart artifacts were removed. In the subsequent preprocessing step, re-referencing was performed, and 1000 ms epochs, including a 200 ms baseline period and an 800 ms interval following stimulus onset, were extracted. Finally, the spherical interpolation of bad channels was carried out for each of the epoched files. An additional low-pass filter at 10 Hz was applied to smooth the ERP waveforms for clearer visualization, before plotting the figures.

The common average reference (AVE) calculation was based on the 37 scalp sites noted above. Both EARS and MM are obtained offline by averaging separate scalp sites (A1 and A2 for the EARS; TP9 and TP10 electrodes for the MM). The L referencing option represents the TP9 electrode, whereas the VERT referencing method represents the Cz electrode. For calculating the REST, we used re-referencing to the REST v1.1 EEGLAB extension [63,64]. The mean number of trials used for grand averages extraction was 53.3 for congruent condition, and 53.5 for incongruent condition (range = 43–59 for congruent condition; range = 41–60 for incongruent condition).

### 2.5. Data Analysis

Data were analyzed using a repeated-measures ANOVA, with post hoc comparisons conducted via Bonferroni-corrected t-tests. All statistical analyses were performed in SPSS (version 26), and data preprocessing was performed in MATLAB (version R2023a), using the EEGLAB toolbox for EEG analysis (version 2024.0). A Greenhouse–Geisser correction was applied to adjust for sphericity violations, and the significance was set at *p* < 0.05. Effect sizes are reported as partial eta squared (*η_p_*^2^).

## 3. Results

### 3.1. Behavioral Results

A high level of accuracy was observed for both the congruent (97.7 ± 1.76%) and incongruent conditions (98.4 ± 1.81%). Although the incongruent condition resulted in longer reaction times, *t* (16) = 2.12, *p* = 0.05, no significant difference in the number of errors, *t* (16) = 1.383, *p* = 0.186, or in the reaction times between the conditions, was detected.

### 3.2. ERP Results

**Figure 2:**
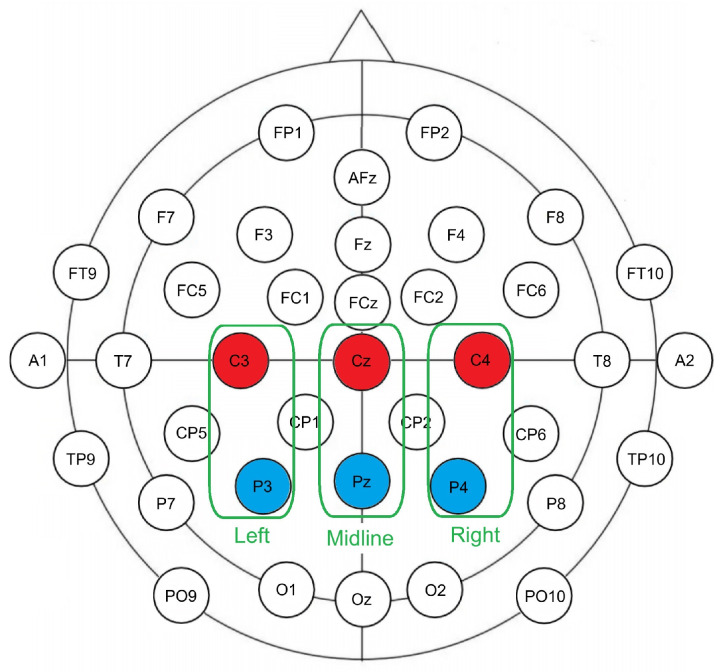
Electrode placement and analysis zones. Green boxes represent laterality grouping: left, midline, and right. Different colors represent frontality grouping: red represents central electrodes, and blue represents parietal electrodes.

As seen in Figure 2, six electrode sites of interest were grouped into five zones, consisting of two bands (frontality) subdivided into three regions (laterality). The first band included central electrodes (C3, Cz, C4), while the second band consisted of parietal sites (P3, Pz, P4). The laterality grouping included three factor levels: left (C3, P3), midline (Cz, Pz), and right (C4, P4). A repeated measures analysis of variance (ANOVA) with within-subject factors of congruency (2), frontality (2), and laterality (3) was conducted, with mean amplitude as the dependent measure in a pre-selected time window of 300–500 ms post-stimulus. The selection of central and parietal sites, as well as the time window, was based on previous N400 research [46]. For the VERT, a 2 × 2 × 2 ANOVA was performed with only two laterality levels, as the midline included the Cz electrode.

**Figure 3:**
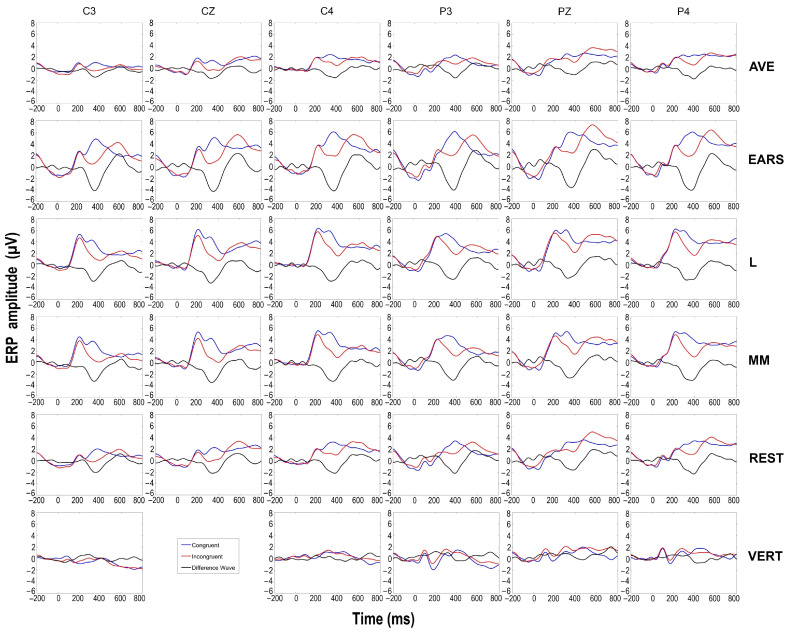
Verification task grand average ERPs in different reference methods. The blue line represents congruent condition, the red line represents incongruent condition, and the black line represents difference wave.

The three-way ANOVA showed a main effect of congruency in all references except the vertex (VERT) (Table 1), with incongruent stimuli eliciting greater negativity than congruent stimuli. The largest effect size was observed with the AVE, F (1, 16) = 19.40, *p* < 0.001, and *η_p_²* = 0.548, followed by the MM, F (1, 16) = 19.16, *p* < 0.001, *η_p_²* = 0.545, the EARS, F (1, 16) = 14.42, *p* = 0.002, *η_p_²* = 0.474, the REST, F (1, 16) = 14.38, *p* = 0.002, *η_p_²* = 0.473, and the L, F (1, 16) = 7.52, *p* = 0.014, *η_p_²* = 0.320. For the VERT, no main effects or interactions were observed. Figure 3 displays the waveforms at central and parietal sites for different references. In all ANOVAs where a main effect of congruency was found, a significant main effect of frontality was also observed, with more positive amplitude values at parietal locations compared to central ones in both conditions.

**Figure 4:**
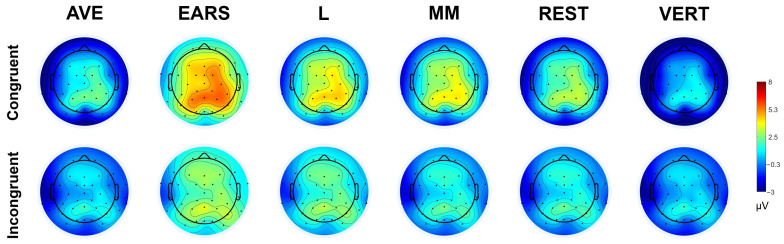
Voltage topographies of congruent and incongruent condition (300–500 ms) in different reference methods.

To examine the differences among the various references in more detail, we conducted a subsequent three-way ANOVA with the factors reference (5), frontality (2), and laterality (3), using difference waves as the dependent measure in a 300–500 ms time window. Since the mean amplitude values in both conditions vary greatly depending on the reference, we chose difference waves as they nullify this effect, providing a less biased measure. The ANOVA revealed a main effect of the reference, F (2.597, 41.555) = 4.43, *p* = 0.011, *η_p_²* = 0.217, indicating that the EARS method produced the largest difference values between the conditions, followed by MM and L, while the REST and AVE methods showed the lowest values of difference waves. Pairwise comparisons revealed significant differences between the REST and EARS referencing methods, *t* (17) = 3.49, *p* = 0.003, and between the AVE and MM referencing approaches, *t* (13) = 3.42, *p* = 0.004, indicating lower values for the REST and AVE amplitudes. The topographic maps shown in Figure 4 for the 300–500 ms time window, based on mean amplitude values, illustrate an overall amplitude shift, while maintaining the same distribution.

**Figure 5:**
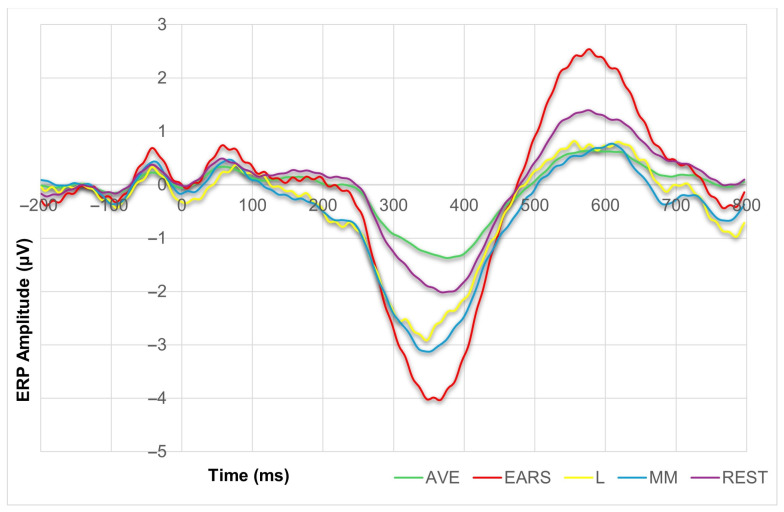
The grand average difference ERPs of six frontal and parietal electrodes (C3, CZ, C4, P3, PZ, P4) for the AVE, EARS, L, MM and REST referencing methods.

To assess the topographic dynamics in more detail, we computed a difference wave, shown in Figure 5, which represented the average of all the electrodes of interest (C3, Cz, C4, P3, Pz, P4) for each of the referencing methods, except for the VERT. We then performed a series of correlations between these difference waves in the 300–500 ms time window. As expected, the Pearson correlation test revealed very high correlations between all of the referencing solutions (Table 2), indicating that the topographic dynamics are most similar between the AVE and REST referencing methods, r = 0.999, df = 52; *p* < 0.001. In contrast, the lowest correlation was found between the AVE and L referencing options, r = 0.945, df = 52; *p* < 0.001.

### 3.3. Statistical Parametric Scalp Mapping (SPSM) Analysis

In the first part of the analysis, we used an a priori selection of six central and parietal electrodes based on previous research to measure the N400 effect with different reference approaches. Since the results showed variation in effect size with different reference methods, we decided not to use a predetermined set of electrodes in the second part of the analysis. Instead, we examined the variation in the N400 effect across all electrodes in all referencing approaches. Rather than an a priori region of interest (ROI) selection, we performed a comparison of average amplitudes between the experimental conditions of all electrodes in the 300–500 ms poststimulus time window for each of the referencing methods.

**Figure 6:**
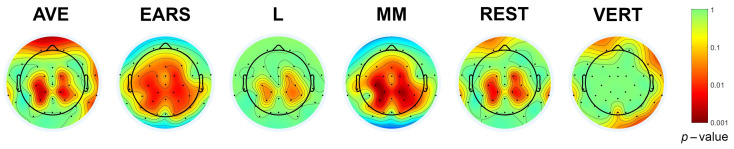
SPSM of different referencing methods for the 300–500 ms time window.

As shown in Table 3, the largest number of statistically significant differences between the two experimental conditions (22 out of 35 electrodes, or 63%) was obtained using the MM, followed by EARS, with 21 out of 35 electrodes (60%), AVE, with 15 out of 37 electrodes (40%), REST, with 13 out of 37 electrodes (35%), VERT, with 11 out of 36 electrodes (31%), and L, with 9 out of 36 electrodes (25%). The SPSM in Figure 6 illustrates the shifts in the scalp distribution of *p*-values, depending on the different referencing methods. This distribution was obtained using EEGLAB’s built-in statistics with FDR correction.

An inspection of the t-values (Table 3) reveals consistent directions in the significant differences, with more negative amplitudes in the congruent condition across all referencing methods except for VERT, which showed a reversed pattern, displaying more positive amplitude values in the incongruent condition. As for the most pronounced effect, its location slightly varies depending on the reference method (Table 3). However, most of the referencing options (AVE, EARS, REST) show the strongest effects at CP2, CP1, and P3 sites, respectively, whereas, in the case of the MM, the order was somewhat different, with P3 showing the strongest effect, followed by CP2 and CP1. For the L reference, the strongest effect was found at the FC2, C4, and P3 electrodes, respectively. Finally, the VERT produced the strongest effects at the TP10, FT10, and F8 sites, respectively, showing the expected opposite direction of difference.

Observing the distribution and the sign of t-values (Table 3), it is evident that all referencing methods except for VERT share a similar trend of difference increasing from lateral to central positions. The VERT shows the opposite pattern, with differences increasing from central to lateral positions. Accordingly, there was no electrode site that exhibited a statistically significant effect across all referencing methods. However, an examination of the effects in referencing methods that produce similar trends (AVE, EARS, L, MM, and REST) showed statistically significant differences between experimental conditions at nine sites that persisted across all the aforementioned referencing methods: FC1, FC2, C3, C4, CP1, CP2, CP6, P3, and P4.

## 4. Discussion

In the current study, the effects of several commonly used offline referencing schemes (AVE, EARS, L, MM, REST, and VERT) were analyzed in a picture–word verification task, based on previous findings regarding the most frequently used references in picture-evoked N400 studies [46]. There has been an ongoing debate about the optimal digital reference choice, with AVE and REST being regarded as the best options, since they are considered the least biased compared to the other references [8,10,11,14,15,16,17,18,19]. On the other hand, research thus far indicates that MM is the most commonly used referencing option, at least in picture-evoked N400 research [46]. In addition to facilitating easier result comparisons, it has been shown that MM avoids hemispheric asymmetry [47] and produces larger N400 amplitudes and effect sizes compared to the AVE and REST approaches [45,48,52].

The results of our study indicate that all referencing options (AVE, EARS, L, MM, and REST), except for VERT, produced a firm N400 effect, with the largest amplitude observed at the centroparietal sites in the picture–word verification task. Considering that the scalp activity of the N400 component is most prominent at the superior central and parietal areas [21], it is expected that the VERT is not a good choice, as its location is significantly influenced by the very activity it is supposed to be free of. Additionally, it produces a reversed pattern compared to other referencing choices, as it attenuates signals from nearby areas while amplifying those from more distant regions. Compared to the findings of Li et al. [45], who reported that the LM produced the largest effect, followed by the REST and AVE, our results differ, with the largest effect being observed using AVE, followed by MM, EARS, REST, and L. However, in both studies, the differences in effect sizes between AVE, REST, and MM (or LM) are quite similar. These discrepancies could be attributed to various factors, such as the number of participants, trials, or differences in signal quality. Additionally, we have seen that L produced the weakest effect size and the fewest number of statistically significant differences across the scalp. The L reference tends to produce smaller N400 effects compared to AVE, REST, or the MM, because it introduces asymmetry in the recorded signals. Since the N400 component has a vertical orientation [52], the L reference can distort signals, particularly those from the central and right hemisphere regions. This asymmetry suppresses the overall signal amplitude and reduces the effect size. In contrast, AVE, REST, and the MM combination provide a more symmetrical reference across the scalp, preserving the full strength of the vertically oriented N400 component, and resulting in larger and more reliable effects.

As for the difference wave, the EARS referencing method produced the largest values between the conditions in the 300–500 ms time window, followed by MM, L, REST, and AVE. However, since EARS also produces the largest standard error, it diminishes the influence on the N400 effect size. Subsequent SPSM analysis also showed that the MM and EARS referencing methods proved to be the least accurate in pinpointing the most optimal N400 effect locations, demonstrating significant differences between the conditions across a large number of recorded sites (63% for MM and 60% for EARS), ranging from frontal to occipital regions. A similar pattern of significant effect distribution using MM was also reported in previous research, compared to the somewhat restricted area of effect produced by AVE [45]. The main reason for this is that the specific localization and bilateral nature of the MM and EARSs allow them to detect subtle effects across a larger number of superior sites compared to the more global approaches of AVE and REST, especially in vertically oriented components such as N400 and P300 [52].

Compared to the other referencing options, AVE and REST produced both significantly lower values of the difference waves, and a fewer number of sites that showed significant differences between conditions. Smaller amplitude values of the ERP components obtained using AVE compared to MM and REST were also confirmed in other studies [15,65], but this finding is still inconclusive, as there is also evidence of the opposite pattern between the same referencing choices [18]. This difference can be partly explained by the different orientations of various ERP components, as it is known that the choice of AVE diminishes the statistical power of vertically oriented ERP components, such as N400 and P300 [52]. Relative to other methods, the AVE and REST referencing approaches emphasize more specific regions of interest, primarily focusing on centroparietal sites. It is important to note that, due to the nature of AVE and REST, there are also regions with reversed effects, as shown in the t-values obtained in our analysis. This finding is consistently observed when using AVE and REST as referencing solutions [18,45]. This polarity reversal occurs because both AVE and REST methods adjust the EEG signal based on overall activity across the scalp. In the case of AVE, the signal is referenced against the average of all electrodes, so strong activity in one region (such as central or parietal areas) can make weaker or opposite signals in other regions (such as anterior or lateral sites) appear reversed. Similarly, REST approximates a neutral “infinity” reference and redistributes the signal across the scalp based on a model of brain activity. This redistribution can also cause polarity shifts, as brain regions with strong activity dominate the reference, leading other areas to show opposite effects. Such polarity reversals, depending on the reference used, must always be considered when interpreting results, especially in the case of AVE and REST, as they can lead researchers to incorrect conclusions if they are not familiar with how these two referencing solutions operate.

In light of our findings, there are at least two practical reasons for choosing AVE and REST as the preferred digital references. First, despite producing the smallest difference waves, AVE and REST demonstrate the best balance between effect strength and precision. Second, AVE and REST referencing options produced the lowest standard error compared to the other referencing option in both conditions, thus maintaining the high effect size despite comparably low amplitude values. Since the standard error of the obtained score is a reliable measure of its precision [66], it is additional confirmation that AVE and REST provide more accurate results compared to the other referencing options.

The analysis of the topography dynamics showed very high correlations between all of the referencing methods. This finding, along with the overall amplitude shift, is in line with the basic principles of re-referencing, which state that it does not affect the topography distribution but influences the amplitude values [4]. An inspection of the effect stability failed to identify sites that produced consistent patterns of difference that could withstand all of the referencing methods used in this study, primarily due to the VERT, as it is itself located in the region of interest. However, the analysis that included all other referencing methods showed a consistency of significant differences between experimental conditions at nine electrode sites, spanning from superior frontocentral to parietal regions.

Our recent studies have investigated ERP components and protocols to identify markers of neurological disorders with potential diagnostic value [55,56,57]. The findings from this study highlight the critical role of reference selection when using ERP amplitude values for diagnostic and clinical classification purposes.

## 5. Conclusions

Results show that all digital references used in this study, except for the VERT, could adequately observe the centroparietal N400 effect in the picture–word verification task. In our study, we obtained the strongest N400 effect using the AVE, whereas the EARS resulted in the largest difference waves. Although AVE and REST referencing methods produced the smallest difference waves, they showed the lowest standard error, thus maintaining both high effect values and precision in the obtained results. Additionally, the SPSM analysis indicated that AVE and REST provided the most precise areas of significant effect distribution. Finally, considering the most pronounced effects across different referencing methods, our study suggests that the most optimal N400 ROI in the picture–word task should include electrodes from the superior frontocentral, central, centroparietal, and parietal sites. Specifically, these should predominantly consist of superior sites, as indicated by Kutas and Federmeier [21], considering the orientation of the N400 effect [52] and the expected lateral effect decline.

## Figures and Tables

**Table 1:** Means, standard errors, and one-way analyses of variance in different referencing methods.

	Congruent		Incongruent		*F*(1, 16)	*η_p_^2^*
	M	SE	M	SE		
AVE	1.77	0.23	0.89	0.17	19.40 ***	0.548
EARS	4.97	0.65	2.68	0.43	14.42 **	0.474
L	3.82	0.67	2.13	0.46	7.52 **	0.320
MM	3.44	0.44	1.48	0.37	19.16 ***	0.545
REST	2.62	0.36	1.42	0.21	14.38 **	0.473
VERT	0.80	0.43	0.72	0.27	0.03	0.002

Note: ** *p* < 0.01; *** *p* < 0.001.

**Table 2:** Correlation matrix between grand average difference ERPs of six frontal and parietal electrodes (C3, CZ, C4, P3, PZ, P4) for the AVE, EARS, L, MM and REST referencing methods.

Reference	AVE	EARS	L	MM	REST
AVE	-	0.981	0.945	0.959	0.999
EARS	0.981	-	0.986	0.995	0.988
L	0.945	0.986	-	0.994	0.956
MM	0.959	0.995	0.994	-	0.968
REST	0.999	0.988	0.956	0.968	-

**Table 3:** (A) *p*-values of *t*-tests conducted between experimental conditions in the verification task for all electrodes using different referencing methods. (B) Values of *t*-tests conducted between experimental conditions in the verification task for all electrodes using different referencing methods.

A	MM	EARS	L	AVE	REST	VERT	B	MM	EARS	L	AVE	REST	VERT
FP2	0.407	0.168	0.833	0.017	0.111	0.025	FP2	−0.852	−1.444	0.215	2.662	1.688	2.471
FP1	0.753	0.675	0.353	0.007	0.03	0.013	FP1	0.32	−0.427	0.957	3.061	2.382	2.78
FT10	0.835	0.702	0.353	0.005	0.055	0.007	FT10	0.212	−0.389	0.957	3.272	2.07	3.113
F8	0.444	0.308	0.896	0.213	0.651	0.012	F8	−0.785	−1.054	−0.133	1.298	0.461	2.821
F7	0.648	0.35	0.915	0.16	0.392	0.046	F7	−0.465	−0.962	0.108	1.475	0.879	2.163
FT9	0.723	0.233	0.675	0.061	0.141	0.032	FT9	−0.361	−1.238	0.428	2.014	1.549	2.349
T8	0.038	0.038	0.142	0.796	0.373	0.269	T8	−2.257	−2.26	−1.543	−0.263	−0.917	1.145
FC6	0.133	0.127	0.399	0.974	0.597	0.098	FC6	−1.585	−1.608	−0.867	−0.033	−0.539	1.756
F4	0.048	0.039	0.138	0.424	0.215	0.525	F4	−2.143	−2.254	−1.56	−0.821	−1.29	0.649
FZ	0.004	0.006	0.058	0.357	0.085	0.267	FZ	−3.34	−3.179	−2.045	−0.948	−1.838	1.149
F3	0.033	0.02	0.184	0.782	0.289	0.262	F3	−2.328	−2.582	−1.388	−0.282	−1.096	1.163
FC5	0.048	0.014	0.265	0.771	0.509	0.108	FC5	−2.143	−2.77	−1.154	0.296	−0.676	1.702
T7	0.042	0.033	0.139	0.354	0.196	0.755	T7	−2.214	−2.337	−1.557	−0.955	−1.349	0.317
FC2	0.001	0.001	0.005	0.001	0.001	0.288	FC2	−4.132	−4	−3.255	−3.834	−3.908	−1.1
FCZ	0.027	0.039	0.169	0.414	0.23	0.446	FCZ	−2.428	−2.249	−1.44	−0.84	−1.248	0.781
FC1	0.002	0.005	0.029	0.026	0.017	0.949	FC1	−3.622	−3.244	−2.406	−2.455	−2.652	0.065
C4	0.002	0.002	0.006	0.03	0.01	0.856	C4	−3.613	−3.636	−3.191	−2.389	−2.904	−0.184
CZ	0.013	0.014	0.088	0.086	0.054	***	CZ	−2.785	−2.77	−1.819	−1.83	−2.078	***
C3	0	0.001	0.011	0.001	0.001	0.981	C3	−4.784	−4.066	−2.874	−4.274	−3.888	0.025
CP6	0.002	0.004	0.015	0.022	0.014	0.838	CP6	−3.702	−3.318	−2.731	−2.545	−2.743	−0.208
CP2	0	0	0.007	0	0	0.274	CP2	−4.896	−4.462	−3.098	−4.627	−4.473	−1.133
CP1	0	0.001	0.008	0	0.001	0.855	CP1	−4.801	−4.128	−3.043	−4.474	−4.087	−0.185
CP5	0.085	0.052	0.292	0.944	0.526	0.119	CP5	−1.835	−2.098	−1.089	0.071	−0.648	1.646
P4	0	0.002	0.01	0.005	0.005	0.611	P4	−4.366	−3.642	−2.913	−3.242	−3.255	−0.519
PZ	0.009	0.009	0.078	0.112	0.049	0.541	PZ	−2.952	−2.972	−1.885	−1.68	−2.132	0.625
P3	0	0.001	0.006	0	0.001	0.938	P3	−5.333	−4.098	−3.16	−4.367	−4.026	0.08
TP10	***	0.966	0.346	0.007	0.052	0.003	TP10	***	0.044	0.971	3.087	2.099	3.552
P8	0.004	0.021	0.109	0.917	0.392	0.201	P8	−3.316	−2.548	−1.696	−0.106	−0.879	1.334
O2	0.009	0.011	0.154	0.726	0.293	0.247	O2	−2.99	−2.894	−1.498	−0.356	−1.087	1.202
O1	0.025	0.059	0.231	0.908	0.499	0.197	O1	−2.468	−2.032	−1.244	−0.117	−0.692	1.345
P7	0.026	0.02	0.085	0.749	0.289	0.274	P7	−2.449	−2.581	−1.836	−0.325	−1.096	1.132
TP9	***	0.106	***	0.133	0.324	0.091	TP9	***	−1.714	***	1.585	1.017	1.798
PO10	0.734	0.344	0.938	0.064	0.256	0.017	PO10	−0.346	−0.976	−0.079	1.986	1.179	2.661
OZ	0.382	0.226	0.824	0.064	0.19	0.018	OZ	−0.899	−1.258	0.227	1.994	1.368	2.629
PO9	0.135	0.069	0.413	0.293	0.786	0.092	PO9	−1.572	−1.951	−0.841	1.088	0.276	1.795
LM	0.476	***	0.127	0.02	0.012	0.016	LM	0.73	***	1.609	2.595	2.848	2.684
RM	0.473	***	0.125	0.007	0.003	0.014	RM	0.735	***	1.618	3.065	3.423	2.752

(A) Orange shading indicates 0.001 ≤ *p* < 0.05, and red shading indicates *p* < 0.001. (B) Blue shading indicates more negative *t*-values, and red shading indicates more positive *t*-values. *** indicates sites used as reference electrodes.

## Data Availability

The datasets used in the current study will be made available from the corresponding author on reasonable request.
